# Agile methods in biomedical software development: a multi-site experience report

**DOI:** 10.1186/1471-2105-7-273

**Published:** 2006-05-30

**Authors:** David W Kane, Moses M Hohman, Ethan G Cerami, Michael W McCormick, Karl F Kuhlmman, Jeff A Byrd

**Affiliations:** 1 SRA International, 4300 Fair Lakes Court, Fairfax, VA 22033, USA; 2 Center for Functional Genomics, Northwestern University, 2205 Tech Drive #2-160, Evanston, Illinois 60208, USA; 3 Memorial Sloan-Kettering Cancer Center, Computational Biology Center, 1275 York Avenue, Box #460, New York, NY 10021, USA; 4 Fred Hutchinson Cancer Research Center, 1100 Fairview Avenue North, Seattle, WA 98109, USA; 5 Applied Biosystems, 850 Lincoln Centre Drive, Foster City, CA 94404, USA; 6 Vanderbilt Medical Center, 2209 Garland Avenue, 416 Eskind Biomedical Library, Nashville, TN 37232-8340, USA

## Abstract

**Background:**

Agile is an iterative approach to software development that relies on strong collaboration and automation to keep pace with dynamic environments. We have successfully used agile development approaches to create and maintain biomedical software, including software for bioinformatics. This paper reports on a qualitative study of our experiences using these methods.

**Results:**

We have found that agile methods are well suited to the exploratory and iterative nature of scientific inquiry. They provide a robust framework for reproducing scientific results and for developing clinical support systems. The agile development approach also provides a model for collaboration between software engineers and researchers. We present our experience using agile methodologies in projects at six different biomedical software development organizations. The organizations include academic, commercial and government development teams, and included both bioinformatics and clinical support applications. We found that agile practices were a match for the needs of our biomedical projects and contributed to the success of our organizations.

**Conclusion:**

We found that the agile development approach was a good fit for our organizations, and that these practices should be applicable and valuable to other biomedical software development efforts. Although we found differences in how agile methods were used, we were also able to identify a set of core practices that were common to all of the groups, and that could be a focus for others seeking to adopt these methods.

## Background

Agile development methods have gained adoption in a wide variety of software development domains [1-5]. To date no group has published a study of agile methods in the context of biomedical informatics in a major bioinformatics journal; indeed, there are few if any articles available describing software development processes used in this application domain. We have used agile development approaches to create and maintain biomedical software. In the process we have observed how well the promises of agile methods fare on our projects and for our users. One of our sponsors, John Weinstein, says that "the success of our programs is attributable in part to our adoption of the agile software development paradigm, which promotes close, iterative interaction between software engineers, biologists, and bioinformaticists" [6]. In this paper we describe our collective and varied experiences with these methods both to foster discussion within the biomedical software community about software process, and to acquaint readers with issues to consider when applying agile methods to biomedical informatics.

We first provide some background on agile methods. We then describe the methods used to conduct this qualitative study of agile practices in software projects at our organizations. We present the results that we gathered, including organizational characteristics and key software development practices. The discussion section addresses our analysis and assessment of these results including similarities and differences among the projects, and lessons learned from using agile methods.

### The Agile Manifesto

The agile movement formally declared its existence in 2000 with the publication of the Agile Manifesto [7]. The practices and methods advocated in the manifesto are not new and have a long history of practice. However, the confluence of new tools, new books, and changes in the developer community has created strong interest in these methods in many software development domains.

The Agile Manifesto describes the principles of agile development. The manifesto does not prescribe a specific methodology or tools, but rather a philosophy for approaching software development. The manifesto reads:

We are uncovering better ways of developing software by doing it and helping others do it.

Through this work we have come to value:

**Individuals and interactions **over processes and tools.

**Working software **over comprehensive documentation.

**Customer collaboration **over contract negotiation.

**Responding to change **over following a plan.

That is, while there is value in the items on the right, we value the items on the left more [7]. (original emphasis)

In this paper, we will illustrate how our interpretation and implementation of these four guiding principles affected our biomedical software development projects.

### Emergent requirements and iterative development

One key assumption underlying agile principles is that requirements for software are emergent, that is, the activity of developing and delivering software yields new understanding of the problem. The pursuit of science is an exploratory process that employs trial and error to find and reject blind alleys among a range of promising options. The idea of emergence contrasts with the more traditional notion that, with enough analysis, all requirements for software development can be understood and documented before the start of programming. Software processes that require scientists to determine detailed requirements up front lead to customer frustration when clinicians and scientists are asked for feature descriptions more precise than they are prepared to give.

The agile view is that the best feedback comes from users interacting with working software. To facilitate this sort of feedback, agile methodologies promote early and frequent delivery of well-tested software. The opportunity to interact early with a program being developed helps users internalize what is possible with software and facilitates a better common understanding of the features needed for project success. By emphasizing early visibility into the software, agile methods are similar to older methodologies like Rapid Application Development (RAD). Agile methods differ from RAD by driving deeper to deliver completed features and by pairing these features with automated tests [8-10]. Unlike traditional development approaches, which can have software development cycles that can last for several months, agile project teams use short cycles that are usually only a few weeks long.

During each of these cycles the software team works through all of the phases of software development – gathering requirements, building code that meets those requirements, testing to ensure that requirements are met, and possibly deploying the code into production for customer use. Customers may choose not to deploy after every iteration, but the software should be stable enough to be deployed after every iteration [11].

## Methods

We began work on this paper by identifying organizations using agile development methods to develop biomedical software. General background on some of the common methodologies is provided in Table 1. The groups were identified during informal discussions at software engineering and bioinformatics meetings and conferences. The groups are summarized in Table 2. This was a convenience sample; it was not randomized. However, we did not select against negative experiences with agile approaches. All but one invitee, who responded too late, joined the group or appointed a representative from their organization.

To capture each project's situation we chose to study qualitatively each project's experience in detail, to try to uncover interesting issues related to the use of agile methods in a biomedical software application domain. We initially conducted a survey of the specific practices used at each organization and assessed the preliminary results. We asked the authors to identify the agile development practices that their respective organizations used. We also gathered context information about each organization. Table 3 summarizes the practices and attributes of each project and the degree to which they were common across these organizations.

We then conducted a second, more detailed survey of the authors' experiences. This survey consisted of a set of mostly open-ended questions [see Additional file 1], which allowed participants to provide details difficult to uncover with more directed questioning. The interviewer followed up each question's answer with additional *ad hoc *questions to clarify interesting details.

We organized the second survey in three sections. The first section covered each biomedical software project's context within its respective organization. We asked participants to describe how the project under study compared with other projects within the organization, when/how/by whom agile methods were introduced, as well as more basic matters such as the duration and current status of the project.

The survey moved on to gather a historical view of the project's software development practices. For each standard project process area (planning, communicating with users, development, testing and deployment) we asked the participant to explain the project's practices and how and why they changed over time, if at all. Some questions focused on details of particular practices highlighted in the agile development literature, such as automated unit testing.

Finally, we presented participants with more open-ended questions. We asked each participant to focus on how the stated values of agile development (collaboration, working software, embracing change, technical excellence and simplicity) contributed to their project experiences. We also asked whether the project used any "non-agile" practices, and whether there were any important issues not covered during the survey interview.

After collecting the responses, we worked through the answers looking both for interesting patterns (either commonalities or differences) among the projects and for noteworthy individual experiences. We followed up with each participant to confirm details if necessary, and incorporated these findings into the paper. When describing specific examples, we do not identify the institution involved. We needed to provide anonymity for potentially sensitive examples and organizations, and for consistency we followed this approach for all examples, sensitive or not.

## Results: multi-site comparison of agile practices

### Context and site characteristics

We studied the agile practices of at least one software development project at each author's organization, summarized in Table 2. The projects we examined were drawn from a diverse set of organizational settings, and represent a wide array of software applications. However, they all shared a number of key characteristics. All of the development teams were fairly small, ranging from one to six people. All of the projects used Java as the primary language for software development. In addition, they all reported that much of the complexity in the software they developed reflected the inherent complexity of biological or clinical study and practice. At four of the six organizations, software development teams were responsible for more than one significant software project at once. Dedicated quality assurance (QA) staff, typical in a corporate software development environment, was absent in all but one project. These characteristics are typical of many other projects and organizations developing bioinformatics software.

There were a number of differences in context among the projects. Development teams came from academic, commercial and government development organizations. The teams built software both for users internal to organizations and users external to these organizations. Two of the organizations used less rigorous development processes before adopting agile methods. For three other organizations, agile development reflected a shift to a more nimble approach from process-heavy software development methods. One organization used agile methods from the beginning of its software development. The projects we studied were not developing safety-critical software, but some were critical to users' daily work or to the launch of a commercial product, while others were public information resources and tools.

One of the major challenges reported by the projects was the need for a close working relationship between at least two different fields: software engineering and biology. Biologists possess a significant amount of specialized knowledge, expressed in an intellectual framework unfamiliar to software developers. When biologists communicate with other biologists, they can frequently assume a mutual level of understanding, allowing them to leave out obvious details during discussions. This tacit knowledge becomes a challenge when communicating with software developers. This information asymmetry surfaced in all of our projects, in both directions. As a simple example, groups reported that sometimes the same terms meant different things to the different groups. In one instance the term, "database", caused confusion – the word meant a relational database system to a developer, but meant a data set to a biologist. Early on during another project, it took several months for developers to realize that mouse pens (the cages that house laboratory rodents) could hold more than one animal at a time.

All of the groups took steps to elucidate crucial but tacit knowledge and to prevent misunderstanding. In addition to *ad hoc *conversations, five of the six groups used a weekly meeting between scientists and bioinformaticians to work through issues. Two groups collocated bioinformaticians and scientists in the same office space to increase the possibility of informal communication, a practice explicitly recommended by some agile methodologies. Other approaches included periodic in-depth interviews with scientists and direct observation of scientists performing daily work. On one project the latter was sometimes tricky, both because investigators were protective of their technicians' work time, and because special clearance or training was required even to be present in some lab space.

The academic tradition of collaborative decision making was common in all of the groups. In this tradition, everyone contributes to the decision-making process. Decisions are made by consensus, when they can be reached. This has the advantage of involving diverse viewpoints and giving everyone a voice, but can sometimes slow decision-making because of conflicting priorities and lack of an authoritative voice to resolve differences.

Sometimes scientists do not reach consensus. Another significant aspect of the academic tradition is that investigators in a lab have a fair degree of independence from one another. Conflicting approaches and research goals can coexist, and individual investigators are accustomed to having the freedom to pursue their own understanding of what is best.

For scientists, software development is an ancillary task in the service of science, rather than a central goal. Software is only valuable if it can enable the otherwise impossible or save valuable time. As a result, some scientists and clinicians are wary of spending too much time on software issues, because this diverts energy away from core scientific projects and publications.

A problem arises, however, when scientist users are not sufficiently invested in the success of a software effort. Any software project requires an effective collaboration of the users of the system, both with each other and with the developers. The decentralized organization of most science labs, where each investigator is free to be as involved or uninvolved as he or she chooses, can make this difficult. For some of our projects, it was difficult to interest users in collaborating closely enough to break down the information asymmetry and to provide a common picture of the user goals for the software effort, leading to mistakes, rework and a slower pace of development. In this way, the biomedical domain can present challenges for agile methods, which explicitly rely on collaboration. On the other hand, agile's focus on collaboration can help organizations give this crucial process appropriate attention.

We believe that both the common characteristics and the range of variation provide a fair cross-section of a significant percentage of biomedical informatics efforts today. This representative context forms the basis for our investigation of the suitability of agile methods for the field.

### Practices

The following sections describe the agile practices our projects used, and include examples of how they were applied.

#### Capturing requirements

Since requirements change often, agile methods avoid detailed written documentation of requirements, in favor of a high-level written description that is "a promise to have a conversation," allowing details to be worked out through verbal communication with the customer. Our projects varied in the level of detail with which they recorded requirements, but none of the groups attempted to describe requirements completely. Two used use cases, and all groups used unstructured text to describe user stories. We all used the combination of short development iterations and customer feedback to resolve the details of specific features. Another feature all projects had in common was the use of web-enabled software tools, such as XPlanner [12], to manage and track requirements. One of the groups had started using simple index cards to track requirements, a practice recommended by many prominent practitioners of Extreme Programming [13], but found that system ceased to work well when a key customer moved to different city.

#### Automated acceptance testing

One group used the acceptance testing process as a central customer collaboration activity. For each feature developed, customers wrote machine-executable acceptance tests whose successful execution confirmed programmers had correctly completed their work. The group used the FitNesse open-source testing tool [14] to organize and execute these tests and included the acceptance tests as part of regression testing.

The process of writing the tests refined and confirmed the users' understanding of their requirements. This exercise helped demonstrate to customers the real complexity of their requirements and the value of their close involvement to getting the behavior right. Just as important, the practice encouraged the scientists and developers in the group to collaborate and to share responsibility for the final result. The key to these accomplishments was convincing the customer that this was best way to ensure the software would perform the business rules correctly – that is, the best way to transfer the users' tacit special knowledge into explicit knowledge used to check the behavior of the software.

#### Open workspaces

All of the groups co-located the developers in a common open workspace. For two groups, customers shared this room as well, so that the proper level of domain expertise was never far away. This arrangement improved communication within the development team and with customers by minimizing barriers and encouraging frequent interactions. When developers had questions about either the software or the customer's requirements, often the person with the answer was in the room and could be queried without the formality and overhead of a full meeting.

#### Project planning and prioritization

Fundamental to all of our projects was the need to accommodate changing requirements. One of the major pain points for our customers in the past, especially for those who had experienced more traditional software methods, was development teams that resisted changes in fundamental assumptions once the project was underway. As discussed above, our customers' exploratory scientific process required that the developers be able to make bold changes in direction. Agile methods provide a number of planning tools to help accommodate change.

### Iteration planning

All of the groups used an iterative approach to organize software development activities. Iterative approaches break up work into smaller pieces, each of which can be measured individually for progress and feedback. This takes place on two levels, the release level and the iteration level. At the release level, the end of each release results in the deployment of software into production. Each release does not take too long, however, so the development team can apply customer feedback coming from use of the working product to iterative refinement. A number of smaller iterations make up each release. It is possible for a release to be composed of only one iteration, so that software is released into production at the end of every iteration.

Each iteration, the development team, working with the customers, plans to implement a set of the customers' high priority features, designs architectural changes, and brings the software to some stable point of completion by the end of the iteration. At the end of each iteration, the development team compares progress to estimates. This comparison can provide early indication if the team is completing functionality more quickly or more slowly than planned.

In our study, the details of the iterative approach varied by project. Four groups had mid-range planning practices that provided course-grained guidance for more than one iteration. One project planned work for several weekly iterations, culminating in a release of software to users. Other projects were organized in "phases" of work, some of which resulted in an internal release for local testing, and some of which resulted in production-quality releases to the public. The lengths of iterations across all projects ranged from one to eight weeks. The iteration lengths for each group are reported in Table 4.

Most groups supplemented iteration planning with shorter range planning. Four groups used daily stand-up meetings (see Table 1). In these meetings developers shared basic status information about their work with each other. This practice provided a synchronized starting point for the group's work each day. Also, managers had the opportunity to hear daily about any obstacles that for some reason had not otherwise come to their attention.

### Estimation

All of the groups used a feature backlog as a central planning tool (again, see Scrum in the sidebar on Common Methodologies). The development group captured all new requests for features and listed them in the backlog where they could be considered for inclusion in future iteration plans. Iteration planning consisted of the selection of features from the backlog for completion in the next iteration. Developers estimated the effort required to complete features, and customers prioritized the most valuable features. When selecting content for an iteration, customers weighed the value of the feature and the effort required to complete it. Once a priority feature set was selected whose required effort fit the available resources, groups varied in how they assigned those tasks to specific developers. In four groups, developers volunteered for specific features and tasks, based on interest or time availability. In one group, a senior developer or architect explicitly assigned tasks to each developer. In one group, there was a single developer, and so all work was implicitly assigned to that developer.

Beyond noting what work was still incomplete, five groups also measured velocity, a simple metric for the amount of work completed during an iteration. To quantify work, our projects used a variety of estimation methods: estimating in pairs, estimating by analogy, estimating by guessing, estimating in ideal days, and estimating in actual time [15,16]. Measuring estimate accuracy using a tracking tool proved helpful for future planning and estimation improvement. These tools calculated velocity, in this case calculated as the amount of work (quantified as estimated effort) developers could complete in a period of calendar time (e.g. a day, or an iteration). The development team used the velocity of past iterations to calculate the customer's budget for selecting features for the next iteration.

### Release planning

One characteristic apparent in our analysis that may be common in biomedical informatics was that our customers generally placed less value on fixed release dates and more value on frequent releases of software. For one project this was not the case, because deliverables had to be synchronized with a larger product release. However, for all other projects, customers wanted to receive frequent improvements to the software but did not care about the precise delivery date, except for important bug fixes. Agile development puts the scope/date tradeoff in the hands of the customer, so that they get to decide whether to include more features in a release and postpone the release date, or release sooner with fewer new features per release. Our customers usually opted for the latter approach. Including smaller amounts of functionality per release also meant that no release could spiral too far out of control, meaning that estimated release dates were reasonably close.

The staffing resources available to our projects were limited, and many of our teams worked on more than one project, often switching without much notice. Iterative development helped by making development estimates and tradeoffs both more accurate and clear to the customer. Notably, our releases were on the shorter side even for agile projects, typically ranging from two to eight weeks. Finally, we remark that even for the project with fixed release dates every three weeks, that project was more reliable in meeting its release dates than similar projects at the same site that did not use agile methods.

### Decision making

In all of the groups, customers regularly took part in requirements prioritization. Furthermore, all of the groups, even those with developers working via a contract, had the flexibility to change requirements and priorities with minimal administrative overhead. Nonetheless, the groups reported a mixed level of success getting project priorities from their customers.

Some groups had a central authority who could weigh conflicting priorities and make a final decision. In other groups, the emphasis on academic freedom and collaborative decision making took first priority, and there was no clear authority to make final decisions. For example, in one project, the customer group was spread among three collaborating institutions. To simplify the decision making process, each institution nominated a representative who spoke for that customer group with full authority. Each representative ranked the feature backlog in order of priority, and the development team averaged these rankings to come up with the final prioritization. The representatives and their organizations accepted the explicit fairness of this approach, and the group was able to achieve consensus on priorities.

Working by collaborative consensus did not, however, work for all our projects. Another group reported a lack of central decision-making authority, internal arguments, conflicting priorities, and slowed development. The group supported a number of different tools, each with different stakeholders. Agile methods alone were not sufficient to bring these stakeholders to consensus.

#### Coding practices

The strongest similarities among the organizations were the methods used to build software. For all of the groups automated testing, refactoring and continuous integration were core practices. While the benefit of these practices is not unique to biomedical informatics, we found it significant that all our projects had resounding praise for the value of these practices. Furthermore, we note that the use of these practices represents greater development discipline and quality than is typical for the field.

#### Automated testing

Automated tests are programs that check software application behavior and report problems. Although only one group used automated acceptance testing, all groups used automated unit testing, and some also automated system tests or load tests. Unlike acceptance tests which were user-oriented, these tests were developer-oriented. Automated unit tests exercise the smallest complete unit of code in the system, typically a method or function. When writing these tests, developers simulate interactions with other portions of the system with stubs or mock objects [17]. Automated system tests exercise the system as a whole, from the user interface or an API to the bottom layers of the system. These tests can leverage the same frameworks as the unit tests, but focus on exercising systems to simulate the software's intended function as a whole. Some groups included user interface testing as part of system testing of web applications, but many of the system tests were driven through programmable interfaces instead. On all projects, automated tests were integrated into a testing framework, such as JUnit [18], that enabled frequent execution. Frameworks such as Cruise Control were then used to enable continuous integration [19]. Some groups scheduled their tests to run every evening. Other groups configured their test frameworks to run their tests whenever changes were checked into their code repositories.

Only one group formally measured test coverage (the percentage of application code exercised by the tests), however all reported that automated tests covered a significant majority of their functionality. All groups felt this sort of coverage was crucial to be able to respond to change as required by our customers, and for finding regression errors early in the release cycle.

#### Refactoring

Refactoring improves the internal design of a unit of code without changing its external behavior [20]. Automated tests are an important prerequisite for refactoring because the unit tests provide the assurance that the external behavior has not changed. The groups studied used refactoring extensively to evolve the design and to improve the readability and maintainability of their software. Several groups reported that before they used automated tests and refactoring, they would sometimes fear modifying legacy code out of concern that something would break. Refactoring and testing created an environment that made code modification easier and more reliable. Many of the groups augmented their ability to refactor by using integrated development environments such as IntelliJ IDEA [21] or Eclipse [22] that included refactoring features.

Refactoring helped our projects maintain simpler code bases that were easier to maintain. One project initially used hash tables for object persistence, working out the business rules and program behavior first. Near the end of the project, the modularity of the code base and the existence of tests allowed the developer to swap in a relational database back end in just a single four-week iteration. Refactoring contributed to the ease of this transition in two ways. First, refactoring done before the change in the persistence layer had helped create a clean interface. Second, the change in persistence layer did not change the behavior of refactored code, and so the existing tests of that layer were used to verify that no errors were introduced. Another group found that combination of automated testing and refactoring made responding to issues raised during code reviews much easier. Before these tools were adopted, code reviews would identify areas for improvement that would have been arduous and time-consuming to implement. With the testing and refactoring tools, implementing the recommendations from code reviews took less time, and resulted in code that was easier to read and to maintain.

#### Pair programming

One of the most controversial practices suggested by Extreme Programming is pair programming [13]. The controversy stems from the difficulty of determining whether doubling up programmers on a programming task saves time because of reduced errors and increased productivity, or wastes time for obvious reasons. Our projects did not use pair programming regularly. Some projects used pair programming in an *ad hoc *manner, e.g. when training new team members, or when working on particularly challenging tasks. Some of the teams were too small for pairing (one developer). In other cases, synchronizing work schedules presented a problem for pairing. Others had past negative experience that led them away from pairing.

### Adoption of Agile Practices

Introducing a new development process can be challenging, even in small groups. Each of the organizations exhibited two characteristics important to the successful introduction of agile methods. Each group had a champion that pushed for the adoption of agile methods, and each group had a sponsor who provided support for the effort [23].

The champions were the ones who initially introduced their respective groups to agile development methods. The champions recommended specific practices and took the lead in helping the teams learn how to apply them.

Because the agile methods for requirements gathering, planning and prioritization change the relationship between customers and software development teams, sponsor support was important to realize this change. Our customers have become some of our biggest proponents. One customer regularly trumpets the success of agile methods when he presents to his peers.

## Discussion

We draw several conclusions from this study. First, agile practices represent a distinct shift from development approaches used previously in our organizations. Second, these methods appear to be particularly valuable to biomedical software projects. Third, groups looking to adopt agile methods should focus on core practices. Fourth, there is room within the principles to adapt practices to local needs.

### Shift from how this community has traditionally approached software

Agile development is not business as usual – it represents a real change in how we develop software in this domain. All of the organizations we studied recognized the difference between their groups now and their groups before adoption of these practices. Many also saw differences between how their teams and their peers in the field developed software. In some cases, agile approaches represent a more disciplined approach to software development. For some in bioinformatics, software is incidental to the scientific results and treated like any other working material, and there is no particular attention to testing or requirements. In other cases, agile approaches reflect a leaner approach to software development than those with more emphasis on modeling, documentation and formal decision-making processes. In both cases, agile encourages deeper collaboration during the development process, with a greater level of shared responsibility.

### Agile methods are valuable for bioinformatics projects

The projects we examined were typical of many in the field today. Staffed with small teams, each needed to create software that could relate to the complexity of biological systems. All of the projects we studied reported many benefits from using agile development techniques. Groups reported improved quality, flexibility, and maintainability. Developers were happier with these practices because they made the dynamic nature of the field more manageable. Because the projects we studied were similar to many others in the field, other groups adopting these methods could experience similar benefits.

Even for projects with different characteristics from the ones in our study, agile methods may still be appropriate. We did not have any large projects in our report. However, there have been reports of successful deployment of agile methods in large-scale projects in other fields [24,25]. Similarly, although our software was not heavily affected by regulatory requirements, there are similar experience reports of agile methods used successfully in regulated fields [26].

#### Adapting to change

Customers' needs in the biomedical domain are characterized by change, sometimes deep and fundamental change. Science is by its nature an exploratory activity. As scientists test hypotheses and gain new knowledge, they uncover new insights and directions for research. For software developers supporting these investigations, this means new requirements. Clinically-oriented biomedical applications are also subject to significant change as the entire health care industry struggles to adopt information technology to improve patient care. Historically this has guided practitioners toward throw-away, quick-and-dirty solutions, or in some medical applications, to avoid software solutions entirely. Agile strikes the right balance by pairing frequent feedback and short iterations with automated testing and refactoring approaches that maintain quality while embracing change. Because agile approaches give developers tools to better manage change, we observed that this approach also made the developers more open to change when required.

#### Enabling collaboration

Biomedical software teams are typically small (especially in academia), and the environment is by necessity collaborative. Biomedical software development projects can require expertise in medicine, biology, statistics and software engineering. The principle of collaboration in the Agile Manifesto in general comes relatively naturally. However, in practice, effective collaboration can be hard to achieve, and agile methods give software development teams tools to collaborate better. The agile methods provide an approach for mediating and prioritizing among the competing interests that can surface among the key stakeholders for particular projects. In our age of high throughput biology, computation has become central to biological inquiry and can no longer be considered ancillary to biological research. Agile methods remind both developers and customers of the importance of shared responsibility and real collaboration for success.

#### Need for software quality

Although the goal of some biomedical informatics projects is to produce high quality operational software, a commitment to software quality has not been historically characteristic of biomedical software not directly involved with patient care. Software is often incidental to scientific publications, and even when software is the focus of a paper, measures of software quality are rarely considered. As a result, quick solutions sufficient to support the research at hand are the rule. However, there is a need for higher-quality software in the biomedical field. First, a focus on quality would give the community greater confidence in the tools that are created. Second, quality would make it easier for groups to share and to extend each other's software. Increasingly, biomedical software projects integrate tools from a wide variety of sources.

The agile practices of continuous integration, automated testing and refactoring make it feasible to modify the software of existing tools without getting bogged down in the legacy code. Without these tools, developers who modify existing code risk breaking features that worked before. When modifying existing software without automated tests they face the unpleasant choice of manually verifying that the previous features still work correctly or skipping such validation and hoping for the best. Many developers react to this choice by frequently cloning existing software rather than modifying it to support new requirements. Heavily cloned software is expensive to maintain and hard to understand, which makes it even harder to modify the existing codebase [27]. However, automated testing and refactoring provide a way to overcome these challenges and to offer a level of quality that has often been missing from academic software.

### Core practices common to all of our projects

Although there are a variety of agile methods and practices, a group of practices was common to all of our groups. These practices are:

• Automated unit tests

• Continuous integration

• Feature backlog

• Refactoring

• Open workspace

Most of these practices (automated unit tests, continuous integration and refactoring) focus on automating the development environment and emphasize the agile principle of working software. Testing, especially automated testing, is anecdotally fairly uncommon within biomedical informatics, but we have found it to be one of the most valuable practices promoted by agile methodologies for this domain. The feature backlog embodies the understanding that there can be an unlimited number of potential requirements with varying degrees of importance and difficulty. The backlog provides a good mechanism to capture these potential features when they surface and to help the customer elucidate and clearly express priorities. The open workspace is useful for facilitating communication within the development team. We believe that other biomedical software development groups adopting or considering agile methods should pay particular attention to these practices. They appear to have broad value and applicability in this domain.

#### There are a variety of ways to apply agile approaches

Every project is different. Even though there were common practices across our projects, the agile principles do not prescribe a specific methodology or set of practices. This freedom was reflected in the variety of approaches used by our groups. Some used use cases, some did not. Some used Scrum meetings, some did not. Groups also varied in the lengths of their iterations. Despite the variation, each group used many practices typical of current agile development approaches and aligned with the four principles outlined in the Agile Manifesto.

### Augmented practices developed

Several projects adopted practices that were not part of the standard agile development portfolio. In some cases, we needed new methods to work with loose collaborations of internal and external groups. In another similar case, a group of developers who supported multiple projects needed better tools to work with the customer to balance priorities among subprojects. In yet other cases, our projects needed new methods to work with participants not co-located with the core development team. Each of the groups responded to these challenges by developing, adapting or adopting additional practices. These additions were still consistent with agile principles and fit with groups' other development practices.

## Conclusion

Agile development methods have gained significant adoption in a variety of software development domains. Our experience demonstrates that many of the benefits of agile documented for other domains extend to biomedical informatics. These methods are well suited to the exploratory, iterative and collaborative nature of scientific inquiry. We have identified a core set of practices common across all of the projects we examined, and we also identified areas where there was significant variation among the projects. We believe that others looking to adopt these methods should focus attention on the common areas identified. The areas in which there is variation are the areas where there is more flexibility to adapt to local needs and circumstances. We have presented our experience using agile development approaches in six biomedical software development organizations. Our projects were typical of many biomedical projects in the field today, and we believe these experiences to be broadly applicable.

## Abbreviations

API Application Program Interface

QA Quality Assurance

RAD Rapid Application Development

RUP Rational Unified Process

XP Extreme Programming

## Authors' contributions

DWK established the initial concept for the manuscript, wrote drafts of the manuscript, edited contributions from other authors, and gathered survey data from his organization.

MMH designed the second, more detailed agile practices survey, interviewed each of the other authors, and collated the responses for the group. He helped place these findings into the paper, and edited drafts of the manuscript.

KFK, JAB, MWM and EGC contributed interview responses for their organizations and helped draft the manuscript.

## Supplementary Material

Additional file 1 Appendix. Survey questions asked about each site. Click here for file

## Figures and Tables

**Table 1:** An overview of two common agile development methodologies, Extreme Programming and Scrum

The principles of the Agile Manifesto are applied in many different software development methodologies. Kent Beck's Extreme Programming is the most well known, but there are many others including Scrum, Crystal, FDD, DSDM [13] [28] [29] [30] [31]. The two methods that were most strongly reflected in our projects were Extreme Programming and Scrum. We briefly describe these methods here.
**Extreme Programming**
Kent Beck's Extreme Programming (XP) is one of the most well known, and also one of the most controversial of the agile methods. Beck took a number of well-known practices and "turned them up to 10" [13]. For example, in XP, peer reviews are implemented as pair programming, i.e. continuous peer review. The method assumes that software is being developed in a dynamic environment, so the software development approach needs to be able to adapt to this rapid change.
XP is organized around short iterations, typically one or two weeks long. Features in XP are described as user stories. Programmers estimate the effort to complete each story. Each iteration the customer gets an effort budget and selects a list of stories for implementation from the list of possible user stories. The customer bases the budget on the completed story velocity from the previous iteration. This means that, iteration by iteration, the customer directs the development team to work on the features most important and immediate to that customer.
The engineering practices of XP are notable as well. The method forgoes a distinct design phase in favor of an evolutionary design approach for the software. The approach focuses on informal communication rather than formal documentation. XP makes extensive use of automated unit and acceptance tests. Programmers run these executable tests throughout the development process. When developing new features, the development team uses "the simplest [design] that could possibly work" [13]. When adding a new feature that requires extension of the existing design, programmers refactor the code first to improve the design, and then add the new feature. Refactoring improves the internal design without changing the external behavior. The automated tests provide verification that the behavior of the code has not changed after refactoring. Developers using XP continually shift back and forth between refactoring activities and new feature development activities. XP uses the combination of testing and refactoring to make the software malleable to accommodate rapid requirements change.
**Scrum**
Scrum is an agile method that focuses on project management [28]. The key practices focus on management of feature backlogs. The mechanics of backlog management are similar to those found in XP, i.e. the customer regularly prioritizes features, and in each iteration the development team implements the top N features from the list. Iterations, called sprints, are 30 days long. In addition to selecting features for each sprint, the development team organizes several sprints into releases. Project managers use "burn down" charts to track progress within a sprint or a release. These charts provide a view of progress by plotting unimplemented features against time remaining in the sprint or release.
Another key practice in Scrum is the scrum meeting. A scrum meeting is a daily stand-up meeting for the development team. In the meeting, each member of the team reports what they did since the last scrum meeting, what work is planned before the next scrum meeting, and obstacles. The meeting is short and facilitates open and frequent communication in the team.
Scrum does not specify a particular engineering approach; the approach says nothing about testing or configuration management practices. Some groups apply XP-style engineering practices to complement their scrum practices, but other variants are common as well.

**Table 2:** A summary of the organizations described in this report

Organization	Type	Application(s) and Users	Team Size	Previous Approach
Applied Biosystems	Commercial	A custom workflow engine as a component to be used by developers of products.	Two developers, a part time project manager, and a customer.	Approach based on the Rational Unified Process (RUP)
Fred Hutchinson Cancer Research Center	Academic	A community tool to collect, analyze, report, and share genetic sequence data.	Four engineers for both developing this application and maintaining legacy systems.	Approach similar to the Rational Unified Process (RUP)
Memorial Sloan-Kettering Cancer Center	Academic	A freely available, open source cancer pathway database with a growing array of public users.	One scientific lead, and one architect/developer.	None. Agile-like practices used since inception of project.
National Cancer Institute	Government (supported by a commercial contractor)	A variety of tools to integrate and visualize integromic data set that are made available to the public.	Three engineers and a bioinformatics analyst.	No explicit process
Northwestern University Center for Functional Genomics	Academic	Two projects written at the Center, with users onsite and at two other institutions participating in a consortium.	Three to five developers, domain and quality assurance staff.	No explicit process

Vanderbilt University Medical Center	Academic	A clinical support application.	Three developers and additional quality assurance and configuration management support staff.	A plan driven development approach that emphasized extensive up front design

**Table 3:** The commonality of key practices and attributes across the projects

Practice\Organization	Applied Biosystems	Fred Hutchinson Cancer Research Center	Memorial Sloan-Kettering Cancer Center	National Cancer Institute	Northwestern University Center for Functional Genomics	Vanderbilt University Medical Center
Customer Collaboration						

Onsite Customer		•		•		
Automated Acceptance Testing						•
Use Cases	•		•			
User Stories	•	•	•	•	•	•

Planning						

Iterative Development	•	•	•	•	•	•
Feature/Product Backlog	•	•	•	•	•	•
Mid-range Planning		•	•	•	•	
Scrum Meeting	•			•	•	•
Developer Task Self-Selection	•	•		•	•	•

Building						

Automated Unit Tests	•	•	•	•	•	•
Collective Code Ownership	•	•		•	•	•
Continuous Integration	•	•	•	•	•	•
Nightly Builds & Tests	•	•	•		•	
Refactoring	•	•	•	•	•	•
Shared/Open Workspace/Team Room	•	•	•	•	•	•

Adoption						

Sponsor	•	•	•	•	•	•
Champion	•	•	•	•	•	•

**Table 4:** Iteration lengths for each project

Organization	Iteration Length
Applied Biosystems	3–5 weeks
Fred Hutchinson Cancer Research Center	2–4 weeks
Memorial Sloan-Kettering Cancer Center	6–8 weeks
National Cancer Institute	2 weeks
Northwestern University Center for Functional Genomics	1–2 weeks

Vanderbilt University Medical Center	4 – 6 weeks
