# Supplementary material for: Agile methods in biomedical software development: a multi-site experience report
Source: BMC Bioinformatics. 2006 May 30;7:273. doi: 10.1186/1471-2105-7-273 (PMC1539031; doi:10.1186/1471-2105-7-273)
Supplement: Additional file 1 — Appendix. Survey questions asked about each site. [file 1471-2105-7-273-S1.pdf]

Appendix

The following is the list of survey questions asked of each author. Each interview took approximately one hour. The interviewer presented the questions over the phone (except when he interviewed himself), so they are not recorded in sufficient detail to stand by themselves as a written interview. Please email the interviewer (moses@moseshohman.com) with any questions.

For any question, if the answer has changed over time, please describe how and why.

1. Describe your project.
2. Describe your users, customers, whatever you call them. If your software is intended for a public audience, describe your user/customer representatives.
3. Did you join the project at the beginning or was the project already in development before you?
4. How long have you been with your project? How long has it lasted overall?
5. When did you introduce agile methods?
6. What existed before the introduction of agile?
7. Were you the main proponent of agile, or were there others? If others, describe.
8. How did you learn about agile?
9. Why did you introduce agile, and in what ways did you introduce it? What problems did you hope agile principles would solve? Did you solve these problems (partially or fully)? Did agile create new problems?
10. Describe your team: how many people, what are their roles. Are these people assigned solely to this project, or do they multitask on multiple projects? If the latter, what percent of their time is devoted to your project?
11. How complex is your software? What is the major source of this complexity (domain, software, management)?
12. How critical is your software and why?

Reminder: For any question, if the answer has changed over time, please describe how and why.

13. How accessible are your customers? Does your customer group have a single voice, that is, is there one person that provides the authority to make decisions when customers disagree with each other?
14. How familiar are your customers with the software development lifecycle?
15. How are features/issues prioritized? In what ways does this work well, and in what ways not so well?
16. How are requirements gathered and tracked? In what ways does this work well, and in what ways not so well?
17. How are completed issues accepted (that is, approved as complete)?
18. How do you estimate how long things will take? How does this vary for bugs versus planned scope? How successful are your estimates? How do you deal with issues that take longer than estimated?
19. Describe your planning processes. Describe ways that agile ideas do not apply to your planning situation.

20. Describe what sorts of planning problems you still have today. Describe hurdles your project environment presents to planning effectively.
21. How often do you release software to your users? Patches vs. larger deployments. Describe changes, as well as any mistakes you may have made.
22. Describe communication with scientists/other customers during: planning, development, acceptance, and maintenance.
23. How do you test your software? How do you protect against regression errors?
24. Do you have a QA process (beta testing) before releasing software? Describe the how and why.
25. What is your automated unit test coverage (tests per public method or percentage of statements executed or some other meaningful measure)? If you have automated functional/acceptance tests, what is the coverage like there?
26. How have your defect rates changed over time, and what are the possible reasons for the change?
27. Does your team have the resources it needs to be fully effective?
28. Does your team reflect on its effectiveness? How often? What changes have you made as a result of these reflection sessions?
29. How and how often do you review code/design/etc?
30. Describe your experience with pair programming, and why you don't do more of it (since we all don't do too much).

For the next five questions, discuss how each principle of agile software affected your project experiences.

31. Embracing Change
32. Collaboration
33. Technical Excellence – do not sacrifice technical excellent, it is just as important as delivering functionality and meeting deadlines
34. Simplicity – the "simplest thing that can possibly work"
35. Working Software – bias for working software over comprehensive documentation

36. Did you do anything "non-agile"? Why did you do it?

The interviewer also presented *ad hoc* follow-up questions during the interview to elucidate interesting issues encountered in the respondents' answers to the above questions.
